# Knowledge of A1c Predicts Diabetes Self-Management and A1c Level among Chinese Patients with Type 2 Diabetes

**DOI:** 10.1371/journal.pone.0150753

**Published:** 2016-03-09

**Authors:** Shengnan Yang, Weimin Kong, Cunyi Hsue, Anne F. Fish, Yufeng Chen, Xiaohui Guo, Qingqing Lou, Robert Anderson

**Affiliations:** 1 Affiliated Hospital of Integrated Traditional Chinese and Western Medicine, Nanjing University of Chinese Medicine, Nanjing, Jiangsu, China; 2 Nursing College, Nanjing University of Chinese Medicine, Nanjing, Jiangsu, China; 3 Jiangsu Province Academy of Traditional Chinese Medicine, Nanjing, Jiangsu, China; 4 Hangzhou No.9 High School, Hangzhou, Zhejiang, China; 5 College of Nursing, University of Missouri-St. Louis, affiliated with the ISP Fellowship Support Program, St. Louis, Missouri, United States of America; 6 Department of Endocrinology, Peking University First Hospital, Beijing, China; 7 Department of Medical Education & Senior Research Scientist, Michigan Diabetes Research & Training Center, Ann Arbor, United States of America; Baylor College of Medicine, UNITED STATES

## Abstract

This study was to identify current A1c understanding status among Chinese patients with type 2 diabetes, assess if knowledge of A1c affects their diabetes self-management and their glycemic control and recognize the factors influencing knowledge of A1c among patients with type 2 diabetes. A multi-center, cross-sectional survey was conducted between April and July 2010 in 50 medical centers in the Mainland China. Participants were recruited from inpatients and outpatients who were admitted to or visited those medical centers. The survey included core questions about their demographic characteristics, diabetes self-management behavior, and A1c knowledge. Overall, of 5957 patients, the percentage of patients with good understanding was 25.3%. In the multivariable logistic regression model, the variables related to the knowledge of A1c status are presented. We discovered that patients with longer diabetes duration (OR = 1.05; 95%CI = 1.04–1.06) and having received diabetes education (OR = 1.80; 95%CI = 1.49–2.17) were overrepresented in the good understanding of A1c group. In addition, compared to no education level, higher education level was statistically associated with good understanding of A1c (*P*<0.001). The percentage of patients with good understanding varied from region to region (*P*<0.001), with Eastern being highest (OR = 1.54; 95%CI = 1.32–1.80), followed by Central (OR = 1.25; 95%CI = 1.02–1.53), when referring to Western. Only a minority of patients with type 2 diabetes in China understood their A1c value. The patients who had a good understanding of their A1c demonstrated significantly better diabetes self-management behavior and had lower A1c levels than those who did not.

## Introduction

With the rapid economic development and lifestyle change in human society, the number of patients with type 2 diabetes is increasing dramatically. A recent worldwide survey conducted in 2013 showed that the prevalence of patients with diabetes aged at 20–79 was 8.3%, and 80% of these patients lived in low- and middle-income countries [1]. In China, it is reported that the estimated prevalence of diabetes is 11.6%, which means 113.9 million Chinese adults had diabetes in 2010 [2]. Development of diabetes-related microvascular and macrovascular complications is associated with metabolic control [3]. The U.K. Prospective Diabetes Study demonstrated that intensive glycemic control could reduce the prevalence of long-term complications [4]. So maintaining stable blood glucose levels as close to normal as possible is the principal goal for diabetes treatment [5]. However, a survey involving 6043 patients carried out in China showed that only 32.1% of them achieved a level of A1c < 7% [6], which is the target set by the China Diabetes Society. This finding demonstrated that most patients did not keep their A1c levels <7%. Glycated Hemoglobin (A1c) is considered the gold standard for glycemic control, because it provides the most objective and reliable information about the past 8–12 weeks glucose control of patients with type 2 diabetes [7]. A1c value is useful in identifying the accuracy of self-glucose monitoring and provides necessary information for patients who do not perform self-monitoring blood glucose (SMBG) [8], which can help guide their diabetes self-management. Testing of A1c has become routine practice, and it has been shown that long-term regular testing of A1c contributes to improved metabolic control [9]. Because of its importance, it makes sense that patients’ knowledge of A1c might be a useful prerequisite for their diabetes self-management [10]. Nevertheless, only about 25% of patients with diabetes truly understand the implications of their A1C value [11].

The American Diabetes Association has launched campaigns to encourage patients with type 2 diabetes to be aware of their target and actual A1c values [12]. Scientific evidence has shown that improving patients’ understanding of their A1c leads to improved diabetes self-management and better glycemic control [11, 13]. However, another study reported a contradictory result that A1c knowledge was not correlated with patients’ diabetes self-management [10]. So it is important to determine whether or not patients’ knowledge of their A1c is indeed associated with their self-management and blood glucose levels. Furthermore, the level of knowledge of A1c among patients with diabetes in China remains to be determined, so it would also be useful to assess Chinese patients’ understanding of A1c.

Therefore, the objectives of this study were to 1) identify current A1c understanding status among Chinese patients with type 2 diabetes; 2) assess if knowledge of A1c affects their diabetes self-management and their glycemic control; and 3) recognize the factors influencing knowledge of A1c among patients with type 2 diabetes in China.

## Materials and Methods

### Patients

A multi-center, cross-sectional survey was conducted between April to July 2010 in 50 medical centers, including 31 administrative divisions (22 provinces, 4 municipalities, and 5 autonomous regions) in the Mainland China. The hospitals were also divided into three regions, which are eastern, central, and western China, based on their economy and geographic location in China [14]. Participants were recruited from inpatients and outpatients who were admitted to or visited those medical centers. Those 50 medical centers included tertiary hospitals, having at least 100 diabetes patients per month during last 12 months. To avoid hospital selection bias, each center needed to cooperate with one or more community hospitals to conduct this survey. Eligible patients signed a written informed consent before participating in the study. The study was approved by the Hospital Ethical Committee of West China Hospital, Medical School Sichuan University.

Eligible patients in the 50 medical centers, identified through electronic medical records and communication with the hospitals, (1) had been diagnosed with type 2 diabetes based on 1999 World Health Organization (WHO) [15] for more than one year; (2) did not have severe diabetes complications, such as end-stage renal disease and heart failure; (3) were mentally healthy enough to answer the questions; and (4) read and understand the questions.

In total, 6043 patients agreed to participate in the study. Eighty-two patients had been diagnosed with type 2 diabetes for less than one year and four patients had incomplete data, therefore these patients were excluded from the study. Finally, the data from 5957 patients were analyzed, of which 3040, 1247, and 1670 were from eastern, central and western china, respectively.

### Data Collection

The study investigators were given detailed instructions on how to administer the study questionnaire during a training program conducted for that purpose. The survey included core questions about their demographic characteristics, diabetes self-management, and A1c knowledge. These questionnaires were distributed to patients who agreed to participate, and when they were returned, we checked the questionnaires to determine if they were filled out completely. We evaluated the patients’ knowledge of A1c by using a simple questionnaire developed for this study. The questions were ‘‘How long does A1c reflect average blood glucose level?” and “What is the recommended A1c level by Chinese Diabetes Society guideline?” Patients were categorized as having a good understanding of A1c if 1) they believed that A1c reflected their blood glucose levels for the past 8–12 weeks; 2) they remembered the ideal value was <7%. Others who had one correct answer and one incorrect answer or had none correct answer were categorized as poor understanding group. In addition, the Summary of Diabetes Self-Care Activities (SDSCA) scale was used to assess patients’ past 7 day’s self-management. The Summary of Diabetes Self-Care Activities (SDSCA) scale was translated into Chinese and its validity and reliability were calculated. Cronbach’s alpha index was 0.773. The scale consists of five dimensions, including diet, exercise, blood glucose testing, foot care, and medication taking.

We determined the following demographic characteristics of the patients: age, gender, education level, income, diabetes duration, how they paid for their medical care, chronic complications and treatment regimens (lifestyle management, oral only, or insulin only, or oral and insulin). We reviewed medical records and latest (i.e. within 3 months) laboratory data to document patients’ fasting plasma glucose (FPG), 2-hour postprandial plasma glucose (2hPPG) and hemoglobin A1c (A1c). All the patients were asked if they had received any diabetes education and the average frequency of SMBG per month at home. Blood glucose was measured with a hexokinase-based method and A1c with high-liquid chromatography at the biochemical laboratories in each medical center.

### Statistical Analysis

SPSS 16.0 was used in all the data analyses. Data were presented as mean (and standard deviation [SD]) if criteria for normal distribution was met or median (25th to 75th percentile) if the criteria for normal distribution was not met. Chi-square test when appropriate was used to compare categorical variables, and two independent-samples *t*-test or Wilcoxon two-sample test when appropriate was used to compare continuous variables. Body mass index (BMI) was calculated as body weight in kilograms divided by squared body height in meters. Diabetes duration was calculated from the date of diagnosis of diabetes. The treatment target of A1c recommended by The China Diabetes Society <7%, was used as a cut point to define good glycemic control in this study.

Logistic regression was performed in order to determine the significant factors, which predicted understanding of A1c. Demographics and clinical factors were selected as independent variables. *P*<0.05 was considered to be statistically significant.

## Results

Patient characteristics are shown by knowledge of A1c level in (Table 1). In the good understanding group, 46.1% were female and the mean age was 60.8 years; in the poor understanding group, 45.7% were female and the mean age was 59.1 years. Mean duration of diabetes in the good understanding group was 10.8 years (±7.3), which was significantly (*P*<0.001) longer than that of 8.1 years (±6.6) in the poor understanding group. Good understanding group had a higher average income and a much higher proportion of patients with education level above high school than those in the poor understanding group (*P*<0.001). More patients in the good understanding group had received diabetes education than those in the poor understanding group (*P*<0.001). There were no differences in BMI, gender, marital status and diabetes complications except coronary arterial disease and hypoglycemia between the two groups.

**Table 1:** Demographic, clinical and biochemical characteristics of study patients (N = 5957).

Variable	Good understanding (n = 1507)	Poor understanding (n = 4450)	*P*-value
	Mean/ number (SD or %)	Mean/ number (SD or %)	
Age, years	60.8 (12.1)	59.1 (12.6)	<0.001
Diabetes duration, years	10.8 (7.3)	8.1 (6.6)	<0.001
BMI, kg/m^2^	24.5 (3.9)	24.5 (4.2)	0.730
SBP, mmHg	129.7 (15.6)	130.3 (17.3)	0.185
DBP, mmHg	78.2 (9.7)	79.2 (10.5)	0.001
Gender			0.805
Male	813 (53.9%)	2417 (54.3%)	
Female	694 (46.1%)	2033 (45.7%)	
Marital status			0.454
Single	36 (2.4%)	82 (1.8%)	
Married	1376 (91.3%)	4080 (91.7%)	
Divorced	18 (1.2%)	45 (1.0%)	
Widowed	73 (4.8%)	238 (5.3%)	
Education level			<0.001
No education level	42 (2.8%)	323 (7.3%)	
Primary school	114 (7.6%)	621 (14.0%)	
Middle school	328 (21.8%)	1181 (26.5%)	
High school	457 (30.3%)	1153 (25.9%)	
Associate degree	293 (19.4%)	644 (14.5%)	
Bachelor and above	273 (18.1%)	528 (11.9%)	
Hypertension	688 (45.7%)	2044 (45.9%)	0.840
Diabetes complications			
Coronary arterial disease	331 (22.0%)	812 (18.2%)	0.002
Stroke	203 (13.5%)	530 (11.9%)	0.112
Nephropathy	217 (14.4%)	675 (15.2%)	0.466
Retinopathy	467 (31.0%)	1387 (31.2%)	0.888
Diabetic foot	69 (4.6%)	263 (5.9%)	0.051
Hypoglycemia	594 (39.4%)	1510 (33.9%)	<0.001
Treatment regime			<0.001
Lifestyle management	50 (3.3%)	171 (3.8%)	
OAD only	482 (32.0%)	1589 (35.7%)	
Insulin only	322 (21.4%)	1075 (24.2%)	
OAD + insulin	653 (43.3%)	1615 (36.3%)	
Received diabetes education	1333 (88.5%)	3419 (76.8%)	<0.001
District in China			<0.001
Eastern	883 (58.6%)	2157 (48.5%)	
Central	291 (19.3%)	956 (21.5%)	
Western	333 (22.1%)	1337 (30.0%)	
Medical fee payment method			<0.001
No insurance coverage	212 (14.1%)	816 (18.3%)	
Insurance coverage	1278 (84.8%)	3545 (79.7%)	
Other	15 (1.0%)	87 (2.0%)	
Monthly income(RMB)	2000 (1500,3000)*	1800 (1100,2800)*	<0.001

Note: *P*-values were derived from 2 independent-samples T test or chi-square test;

*Median (25th percentile to 75th percentile) and their *P*-values were derived from two-sample wilcoxon test;

Abbreviations: BMI, body mass index; BP, blood pressure; OAD, oral anti-diabetic drug.

Overall, the percentage of patients with good understanding was 25.3%. Patients in good understanding group had significantly lower FBG and 2hPPG, and were more likely to use SMBG than those in the poor understanding group. They reported higher average self-care scores in the prior 7 days than the poor understanding group. Additionally, a higher proportion of patients who had well-controlled A1c were observed in the good understanding group (Table 2).

**Table 2:** Univariate analysis of the relationships between understanding of HbA1c, blood glucose and self-management behavior (N = 5957).

Variable	Good understanding(n = 1507)	Poor understanding(n = 4450)	*P*-value
	Mean/ number(SD or %)	Mean/ number (SD or %)	
FPG, mmol/L	7.9 (2.9)	8.2 (3.1)	<0.001
2hPPG,mmol/L	11.0 (4.1)	11.7 (4.5)	<0.001
Diet	18.7 (5.5)	17.6 (6.2)	<0.001
Exercise	9.0 (4.7)	8.0 (5.2)	<0.001
Foot	8.3 (5.3)	6.1 (5.5)	<0.001
Medication taking	6.2 (2.1)	5.9 (2.3)	<0.001
Blood glucose testing	5 (2,10)*	4 (1,12)*	0.090
SMBG frequency monthly	8 (4,14)*	5 (2,10)*	<0.001
HbA1c	7.88 (1.91)	8.43 (2.33)	0.039
<7	356 (23.6%)	761 (17.1%)	
≥7	672 (44.6%)	1690 (38.0%)	
Missing data	479 (31.8%)	1999 (44.9%)	

Note: *P*-values were derived from 2 independent-samples T test or chi-square test;

*Median (25th percentile to 75th percentile) and their *P*-values were derived from two-sample wilcoxon test;

Abbreviations: HbA1c, glycated hemoglobin; SMBG, self-monitoring of blood glucose.

In the multivariable logistic regression model (Table 3), the variables related to the knowledge of A1c status are presented. We discovered that patients with older age (OR = 1.01; 95%CI = 1.00–1.01), female (OR = 1.18; 95%CI = 1.03–1.35), longer diabetes duration (OR = 1.05; 95%CI = 1.04–1.06), and having received diabetes education (OR = 1.80; 95%CI = 1.49–2.17) were overrepresented in the good understanding of A1c group. In addition, compared to no education level, higher education level was statistically associated with good understanding of A1c (*P*<0.001). The percentage of patients with good understanding varied from region to region (*P*<0.001), with Eastern being highest (OR = 1.54; 95%CI = 1.32–1.80), followed by Central (OR = 1.25; 95%CI = 1.02–1.53), when referring to Western.

**Table 3:** Logistic regression for factors associated with good understanding of A1c (N = 5957).

Variable	Mean/ number (SD or %)	OR(95%CI)	*P*-value
Age, years	60.8 (12.1)	1.01 (1.00–1.01)	0.038
Gender: female versus male	694 (25.4%) versus 813 (25.3%)	1.18 (1.03–1.34)	0.016
Diabetes duration, years	10.8 (7.3)	1.05 (1.04–1.06)	<0.001
Education level			<0.001
No education level	42 (11.5%)	Reference	
Primary school	114 (15.5%)	1.20 (0.80–1.78)	
Middle school	328 (21.7%)	2.00 (1.40–2.87)	
High school	457 (28.4%)	2.79 (1.95–3.98)	
Associate degree	293 (31.3%)	3.31 (2.28–4.81)	
Bachelor and above	273 (34.1%)	3.60 (2.46–5.26)	
District in China			<0.001
Eastern	883 (29.0%)	1.54 (1.32–1.80)	
Central	291 (23.3%)	1.25 (1.02–1.53)	
Western	333 (19.9%)	Reference	
Received diabetes education (yes versus no)	1333 (28.1%) versus 174 (14.4%)	1.80 (1.49–2.17)	<0.001

OR, odd ratio; CI, confidence interval

## Discussion

This multi-center study included 5957 patients and was designed to assess knowledge of A1c and its relationships with diabetes self-management and A1c level among Chinese patients with type 2 diabetes. Additionally, we explored the factors associated with higher levels of knowledge about A1c. The sample size in the survey was large and covered all of mainland of China, with exception for Tibet and Hainan Island.

Relatively few participants, i.e. only 25.3% in our study had a good understanding of their A1c. This rate corresponds to a UK study, in which 26.5% of the patients were considered as having a good understanding of A1c [11], and a US survey reported that 25% of their patients with good understanding of A1c [10,16]. These findings indicate that a poor understanding of A1c is common. However, Skeie et al. [17] reported that 58% of the patients they studied had good perceived knowledge of A1c and over 80% of patients knew their last and target A1c value. However, the sample in this study was recruited from just one diabetes outpatient clinic and just contained individuals with type 1 diabetes. What’s more, the sample was relatively small, all of which limit it’s generalizability.

According to the factors associated with good understanding of A1c among patients with type 2 diabetes (Table 3), the study suggests that older patients and patients with longer diabetes duration are more likely pay attention to their diabetes care and accumulate a significant amount of diabetes related knowledge over the years [17]. This study indicates the need to intensify the education for the newly diagnosed patients [18]. More females than males take part actively in their diabetes care as evidenced by Zhangying et al [19], which could account for the finding that females showed a higher rate of understanding their A1c value. Education level is also a positive predictor of good understanding of A1c, which is consistent with a previous study [10], and suggests that patients with a higher education level are more likely to take actions to better manage their diabetes when they understand the implications of an elevated A1C than patients with a lower education level [18]. Studies indicate that patients in the eastern region in China were better educated and were treated in a more detailed and comprehensive method when they were in hospitals. The diabetes educators in this region are trained scientifically and professionally and the medical services in the eastern region are of relatively of good quality [20, 21]. This would explain why patients in the eastern region demonstrated a better understanding of A1c than patients from the central and western regions. Patients who received diabetes education were more likely to have a good understanding of A1c; this finding indicates that diabetes education plays an important role in diabetes self-management, which is also supported by a recent systematic review and other articles [18, 22, 23]. In summary, the findings of the current study regarding the association of multiple factors and the patients’ understanding of A1c need to be confirmed by other studies.

The results also show that there was a correlation between understanding of A1c, diabetes self-management behavior and glycemic control. This implies that a good understanding of A1c can improve clinical outcomes, such as FBG, 2hPPG and A1c. What’s more, patients with good understanding of their A1c performed better self-management behavior. They followed the regimens (i.e. diet, exercise, medication taking and foot care) in the past 7 days more frequently than the patients with a poor understanding of their A1c.

We also found that the average number of days patients performed SMBG in the prior 7 days or the SMBG frequency monthly were significantly related to the understanding of A1c status (Table 2). The establishment of a relationship between understanding of A1c, self-management and clinical outcomes was shown to be significant. This result also fits with another study [11]. Our findings also showed that better glycemic control is achieved among patients who had a good understanding of their A1c, compared to the poor understanding group, is in line with other study findings, which have demonstrated that good understanding of A1c can predict A1c values [11]. A previous study [10] found no correlation between A1c knowledge and self-management behavior. This study inferred that variables other than knowledge play an important role in influencing patients’ diabetes self-management behavior level [11]. However, that study had a small sample and enrolled different races in US, which may have confounded the results.

These findings indicate the importance of providing patients with diabetes clear and specific knowledge about clinical test results, such as their A1c. In actual clinical settings, physicians and other health care professionals should pay more attention to helping patients understand the implications of A1c results rather than just telling patients their A1c values. There are some effective strategies to improve patients’ knowledge of their A1c when patients are educated. For example, in Japan, a diabetes passport is used to record patients’ A1c levels and an information card is attached to the diabetes passport to enable patients to realize how well their glycemic levels are controlled based on their A1c value [24]. In addition, the passport includes clear graphical representations of A1c values [25] like faces whose emotion reflected current level of glycemic control or letter grades ranging from A to F [26]. These strategies may play an important role in helping patients with low educational level understand clinical information and encourage them to record and track clinical data in their diabetes logbooks [27].

Our study has some limitations. First, besides knowledge, there are many other factors influencing self-management behavior and A1c level, such as attitude, self-efficacy [11, 28]. But, we did not measure these variables in this study. Second, we just asked patients two questions to judge their understanding of A1c levels, and did not measure whether they knew their last A1c value and have their A1c test as often as is recommended [10,18]. These aspects may be also important for patients to achieve satisfactory diabetes control. Third, the way we assessed if patients received diabetes education was not specific. We asked them, “Have you received diabetes education before?” Therefore, a “yes” answer might mean very brief instruction during a physician visit rather than structured or formal diabetes education. Finally, there were some missing data for A1c values at each of the 50 study sites likely because sometimes patients forgot to get the blood drawn or, perhaps, they could not afford to get the blood drawn. The missing data must be taken into account when interpreting study findings. But a greater presentation in the poor understanding group (80.6%) than another group among patients who did not check their A1c levels.

## Conclusions

Only a minority of patients with type 2 diabetes in China understood their A1c value. The patients who had a good understanding of their A1c demonstrated significantly better diabetes self-management behavior and had lower A1c levels than those who did not. Our study found that patients’ understanding of their A1c was associated with age, gender, education level, diabetes duration, regions and diabetes education. These findings revealed the need for more effective diabetes education and care, and indicate the importance of HCPs actively communicating their A1c test results with patients and insuring that their patients understand the meaning of their A1c level. Increasing patients’ understanding and recognition of their clinical test information, including their A1c, would help patients effectively manage their diabetes.
